# Clinical features and prognosis of isolated cardiac sarcoidosis diagnosed using new guidelines with dedicated FDG PET/CT

**DOI:** 10.1007/s12350-022-03034-0

**Published:** 2022-07-08

**Authors:** Tomohisa Okada, Naoto Kawaguchi, Masao Miyagawa, Marika Matsuoka, Rami Tashiro, Yuki Tanabe, Tomoyuki Kido, Toru Miyoshi, Haruhiko Higashi, Takeshi Inoue, Hideki Okayama, Osamu Yamaguchi, Teruhito Kido

**Affiliations:** 1 Department of Radiology, Ehime University Graduate School of Medicine, Shitsukawa, Toon, Ehime 791-0295 Japan; 2 Department of Cardiology, Pulmonology, Hypertension and Nephrology, Ehime University Graduate School of Medicine, Toon, Japan; 3 Department of Radiology, Ehime Prefectural Central Hospital, Matsuyama, Japan; 4 Department of Cardiology, Ehime Prefectural Central Hospital, Matsuyama, Japan

**Keywords:** Isolated cardiac sarcoidosis, prognosis, fluorodeoxyglucose positron emission tomography, computed tomography, cardiac sarcoidosis

## Abstract

**Background:**

Diagnostic guidelines for isolated cardiac sarcoidosis (iCS) were first proposed in 2016, but there are few reports on the imaging and prognosis of iCS. This study aimed to evaluate the use of ^18^F-fluorodeoxyglucose positron emission tomography/computed tomography (FDG PET/CT) imaging in predicting iCS prognosis.

**Methods and results:**

We retrospectively reviewed the clinical and imaging data of 306 consecutive patients with suspected CS who underwent FDG PET/CT with a dedicated preparation protocol and included 82 patients (55 with systemic sarcoidosis including cardiac involvement [sCS], 27 with iCS) in the study. We compared the FDG PET/CT findings between the two groups. We examined the relationship between the CS type and the rate of adverse cardiac events. The iCS group had a significantly lower target-to-background ratio than the sCS group (*P* = 0.0010). The event-free survival rate was significantly lower in the iCS group than the sCS group (log-rank test, *P* < 0.0001). iCS was identified as an independent prognostic factor for adverse events (hazard ratio 3.82, *P* = 0.0059).

**Conclusion:**

iCS was an independent prognostic factor for adverse cardiac events in patients with CS. The clinical diagnosis of iCS based on FDG PET/CT and new guidelines may be important.

**Supplementary Information:**

The online version contains supplementary material available at 10.1007/s12350-022-03034-0.

## Introduction

Sarcoidosis is a systemic granulomatous disease of unknown etiology. The prevalence of cardiac sarcoidosis (CS) is 20–27% among all patients with systemic sarcoidosis in the United States,^1–3^ but this prevalence may be as high as 58% in Japan.^4^ The clinical symptoms of CS include complete atrioventricular block (AVB), ventricular arrhythmias, congestive heart failure, and sudden death.^3^ Sarcoidosis affects the myocardial tissue by inducing inflammatory cell infiltration, granuloma formation, and scarring.^5^ Further, 77% of deaths in patients with sarcoidosis in Japan were reported to be associated with CS.^6^ Corticosteroid therapy is considered to improve long-term prognosis more in patients with higher left ventricular ejection fraction (LVEF) than in patients with lower LVEF, suggesting that early intervention after the detection of cardiac involvement may improve prognosis.^7,8^

Isolated cardiac sarcoidosis (iCS) is CS with no sarcoid involvement in other organs. The diagnosis of iCS requires pathological confirmation by endomyocardial biopsy (EMB). However, the sensitivity of EMB for diagnosing CS is approximately 20%,^9^ and as low as 10% in patients with suspected iCS.^10^ Therefore, the diagnosis of iCS is difficult.

Diagnostic guidelines for iCS were first proposed by the Japanese Circulation Society (JCS) in 2016.^10^ As a prerequisite, patients should be confirmed not to have clinical findings of sarcoidosis affecting other organs. The diagnosis of iCS may be confirmed when non-caseating epithelioid granulomas are found in the myocardial tissues. Patients with clinical findings suggestive of iCS in whom myocardial biopsy is impossible or does not reveal the presence of granulomas in myocardial tissues should be evaluated according to these diagnostic guidelines. Patients who meet at least four of the five major criteria relating to cardiac findings in the “Diagnostic guidelines for cardiac sarcoidosis” (Online Resource 1) are diagnosed with iCS according to the clinical diagnosis group criteria.^10^ Histological evidence of granulomatous inflammation in at least one organ is needed for a diagnosis of CS according to the World Association of Sarcoidosis and Other Granulomatous Disorders or the Heart Rhythm Society 2014 guidelines.^11,12^ These guidelines advocate a group of clinical diagnoses that do not necessarily require a pathological diagnosis of the myocardium. ^18^F-fluorodeoxyglucose (FDG) positron emission tomography (PET) imaging is particularly important, and thus, is included in the mandatory criteria of these guidelines.

There have been few comprehensive reports of iCS and particularly few reports on the imaging and prognosis of iCS diagnosed using the JCS 2016 guidelines. Therefore, this study aimed to evaluate the FDG PET/computed tomography (CT) imaging findings and prognosis of patients with iCS.

## Methods

### Patients

The clinical and imaging data of a total of 306 consecutive patients with clinically suspected CS or known extracardiac sarcoidosis who underwent FDG PET/CT with a dedicated preparation protocol (long fasting protocol) in the Ehime University Hospital or Ehime Prefectural Central Hospital between April 2009 and March 2020 were retrospectively reviewed and analyzed. Findings suggestive of CS included the presence of high-grade AVB or sustained ventricular tachycardia (VT), abnormal ventricular wall anatomy such as basal thinning of the ventricular septum, unexplained heart failure or focal ventricular wall asynergy, presence of late gadolinium enhancement (LGE) on cardiovascular magnetic resonance (CMR), and presence of suspected extracardiac sarcoidosis lesions.

The inclusion criteria were as follows: (1) diagnosis of CS according to the Guidelines for Diagnosis and Treatment of Cardiac Sarcoidosis by the JCS in 2016, (2) presence of abnormal FDG uptake, and (3) patients who were followed up for ≥ 6 months after FDG PET/CT imaging. The exclusion criteria were as follows: (1) patients who had started corticosteroid therapy or other immunosuppressive therapy before FDG PET/CT imaging and (2) patients with a history of myocardial infarction. Finally, 82 patients were included in this study. They were classified as having iCS or systemic sarcoidosis including cardiac involvement (sCS) based on the guidelines for the diagnosis and treatment of cardiac sarcoidosis proposed by the JCS in 2016. The study protocol conformed to the ethical guidelines of the Declaration of Helsinki and was approved by the ethics committees of Ehime University Hospital (approval number: 2003010) and Ehime Prefectural Central Hospital (approval number: 29–97). The requirement for informed consent was waived owing to the retrospective nature of the study.

### Image acquisition and analysis

To suppress physiological myocardial FDG uptake, we used the dedicated preparation protocol: we instructed the patients to eat a low-carbohydrate (< 5 g) diet and thereafter fast for ≥ 18 h before FDG injection.^13,14^ The median fasting time was 19.5 (18.5–20.0) h. Whole-body PET/CT scans were obtained 60–90 min after the intravenous administration of 3.0–3.7 MBq FDG/kg body weight using a multi-slice scanner (Discovery 600 or Discovery ST Elite, GE Healthcare, USA).

The PET/CT images were analyzed in consensus by two experienced radiologists (6 and 14 years of experience in PET image reading) who were blinded to the clinical data. We classified myocardial FDG uptake as none, diffuse, focal, and focal-on-diffuse patterns. Focal and focal-on-diffuse patterns were defined as abnormal FDG uptake.^13^ We also evaluated the presence of abnormal regional uptake in the left ventricular (LV) wall according to the 17-segment model^15^ by referring to the polar map display created by the modified Aladdin software on the Xeleris workstation (GE Healthcare, USA). We also evaluated the presence of right ventricular (RV) wall uptake. We set the volumes of interest in the whole LV myocardium and descending aorta, and measured the myocardial maximum standardized uptake value (SUVmax) and mean SUV (SUVmean) of the blood pool.^16^ We calculated the target-to-background ratio (TBR: myocardial SUVmax/blood pool SUVmean).^17^

### Follow-up

Primary adverse events included cardiac death and fatal ventricular arrhythmia (FVA), including sustained VT, ventricular fibrillation (VF), and cardiac arrest, and hospitalization for heart failure. FVA included those observed on the implantable cardioverter-defibrillator or cardiac resynchronization therapy defibrillator. The time interval to the onset of adverse events was measured from the date of the FDG PET/CT scan. Patients were monitored for the occurrence of adverse events through December 2020. During follow-up, patients were regularly examined by a cardiologist, and echocardiography and electrocardiography were performed as appropriate at the cardiologist’s discretion.

### Statistical analyses

Data are expressed as the median (interquartile range) or n (%). Welch’s t test or the Wilcoxon rank-sum test was used to compare continuous variables, and the chi-squared test was used to compare categorical variables. Cumulative event-free survival between the iCS and sCS groups was analyzed using the Kaplan–Meier method and log-rank test. Univariate and multivariate Cox proportional hazards models were used to analyze the risk of adverse events associated with selected variables. Variables that were statistically significant according to the univariate Cox proportional hazards model were selected for inclusion in the multivariate model. The number of variables in the multivariate Cox proportional hazards model was set to two because of the limited number of events. Statistical analyses were performed using JMP (version 14.2) statistical software (SAS Institute, Cary, NC, USA). Statistical significance was set at* p* < 0.05.

## Results

### Patient characteristics

**Figure 1:**
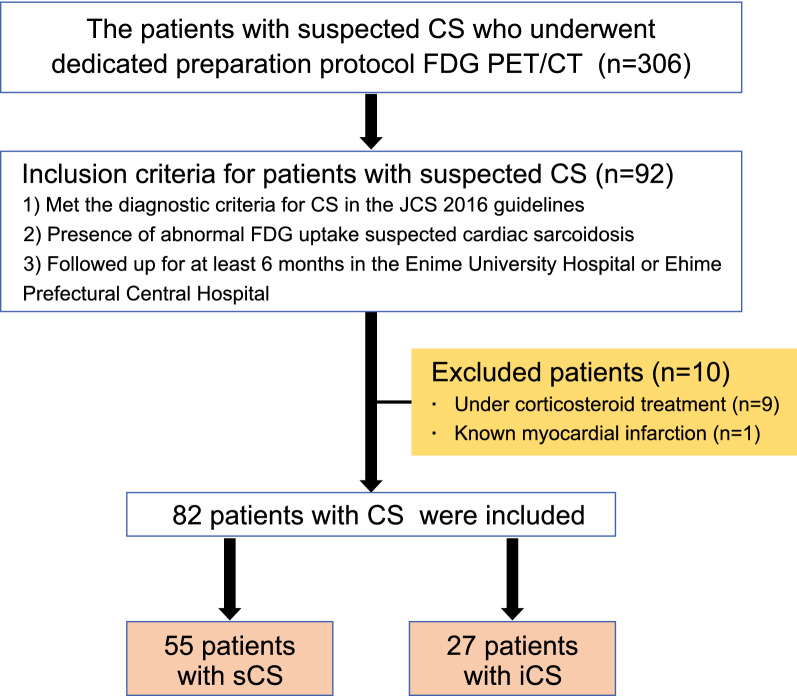
Flowchart of study enrolment. *CS*, cardiac sarcoidosis; *FDG*, ^18^F-fluorodeoxyglucose; *PET*, positron emission tomography; *CT*, computed tomography; *JCS*, Japanese Circulation Society; *iCS*, isolated cardiac sarcoidosis; *sCS*, systemic sarcoidosis with cardiac involvement

The flowchart of patient enrolment is shown in Figure 1. Patients who had started corticosteroid therapy before FDG PET/CT imaging (n = 9) or those with known myocardial infarction (n = 1) were excluded. Therefore, 82 patients were included in the study. Fifty-five patients (67.1%) were classified as having sCS and the remaining 27 patients (32.9%) as having iCS according to the JCS 2016 guidelines. FDG PET/CT follow-up for evaluation of extracardiac lesions was performed in 8 patients in the iCS group, and none of them showed any evidence of extracardiac lesions.

The clinical characteristics of the patients are summarized in Table 1, and the characteristics of patients with iCS are presented in Online Resource 2. There were significant differences between the iCS and sCS groups in terms of sex, angiotensin-converting enzyme (ACE) levels, soluble interleukin-2 receptor (sIL-2R) levels, LVEF, the frequency of LVEF < 50%, number of major criteria met, the positive rate of EMB, the New York Heart Association functional classification class, and the frequency medical therapy for heart failure.

**Table 1:** Patient characteristics

	sCS (n = 55)	iCS (n = 27)	*P* value
Male	15 (27.3%)	19 (70.4%)	0.0002
Age (years)	62 (57–70)	64 (59–69)	0.82
*Blood test*			
ACE (IU/L)	15.2 (11.9–21.0)	10.4 (7.6–12.8)	< 0.0001
Lysozyme (μg/mL)	8.5 (7–10.2)	7 (5.4–9.7)	0.052
sIL-2R (U/mL)	493.5 (412.3–680.3)	288.5 (220.5–365)	< 0.0001
CRP (mg/dL)	0.1 (0.04–0.18)	0.14 (0.04–0.35)	0.40
BNP (pg/mL)	95.1 (48.0–240.2)	239.3 (52.3–412.5)	0.12
*Electrocardiography *			
Advanced atrioventricular block	22 (40%)	7 (25.9%)	0.21
Fatal ventricular arrhythmia	14 (25.5%)	9 (33.3%)	0.46
*Echocardiography*			
LVEF	52 (34–63)	41 (34–50)	0.018
Decreased LVEF < 50%	24 (43.6%)	20 (74.1%)	0.0094
Basal thinning of interventricular septum	7 (12.7%)	8 (29.6%)	0.063
Number of major criteria met^a^	4 (3–4)	4 (4–5)	0.0039
Defibrillator implantation^b^	19 (34.5%)	11 (40.7%)	0.58
*NYHA functional classification*			0.045
Class I	34 (61.8%)	9 (33.3%)	
Class II	13 (23.6%)	8 (29.6%)	
Class III	4 (7.3%)	7 (25.9%)	
Class IV	4 (7.3%)	3 (11.1%)	
Medical therapy for heart failure^c^	21 (38.2%)	23 (85.2%)	< 0.0001
*Cardiovascular magnetic resonance*	n = 44	n = 25	*P* value
Presence of LGE	43 (97.7%)	25 (100%)	0.45
*Endomyocardial biopsy*	n = 26	n = 21	*P* value
Positive/non-specific findings	5 (19.2%) / 21	0 (0%) / 21	0.034

Data are presented as median (interquartile range) or n (%)

*sCS*, systemic sarcoidosis with cardiac involvement; *iCS*, isolated cardiac sarcoidosis; *ACE*, angiotensin-converting enzyme; *sIL-2R*, soluble interleukin-2 receptor; *CRP*, C-reactive protein; *BNP*, brain natriuretic peptide; *LVEF*, left ventricular ejection fraction; *NYHA*, New York Heart Association; *LGE*, late gadolinium enhancement

^a^Major criteria are in the “Diagnostic guidelines for cardiac sarcoidosis”^10^

^b^Defibrillator implantation includes implantable cardioverter-defibrillator, cardiac resynchronization therapy defibrillator at baseline

^c^Medical therapy for heart failure includes β-blocker, ace inhibitor, angiotensin receptor blocker, mineralocorticoid receptor antagonist, diuretic therapy, and cardiotonic drug at baseline

EMB was performed in 21 patients with iCS, and all of them exhibited negative biopsy results. However, 17 patients (81.0%) had non-specific inflammation, fibrosis, and/or myocardial degeneration, which do not rule out CS. In addition, 8 patients (38.1%) met the minor criteria for CS (monocyte infiltration and moderate or severe myocardial interstitial fibrosis). There were no specific findings for other diseases such as hypertrophic cardiomyopathy, amyloidosis, Fabry disease, or eosinophilic myocarditis. CMR was performed in 25 patients (92.6%) in the iCS group, all of whom had LGE, suggesting a CS pattern.^18,19^ In the two patients who did not undergo CMR, follow-up PET/CT was conducted. One patient was receiving corticosteroid therapy and confirmed with decreased cardiac FDG uptake on the follow-up PET/CT. The other was not receiving corticosteroid therapy, and follow-up PET revealed mixed findings (decreased and increased FDG uptake).

### Image interpretation of FDG PET/CT

**Figure 2:**
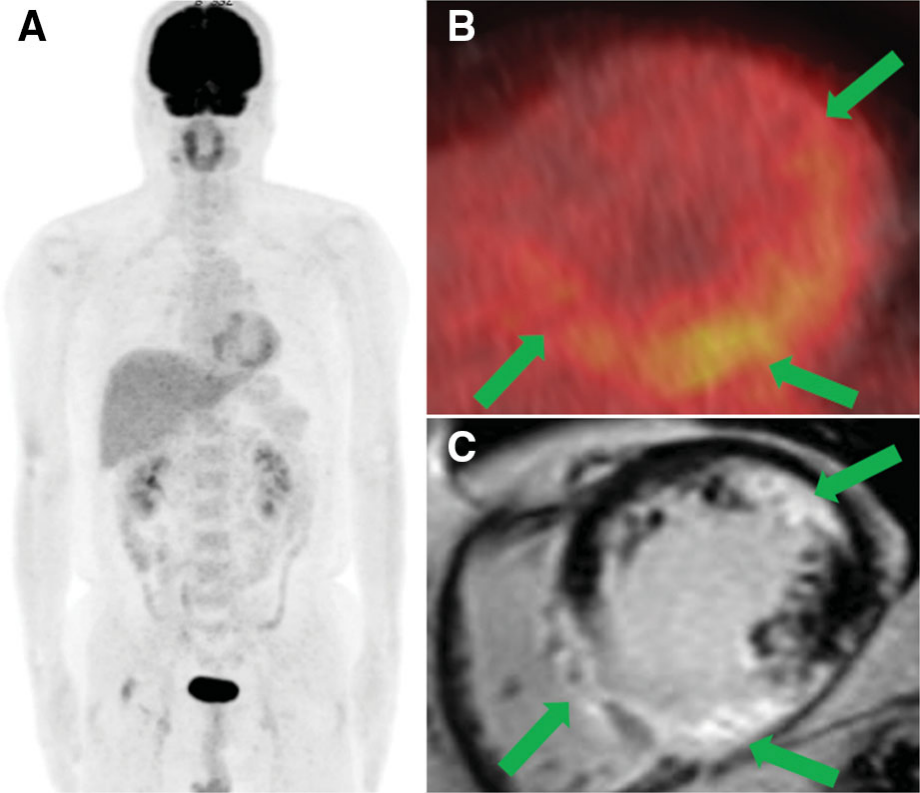
Images of a representative case of isolated cardiac sarcoidosis. A 53-year-old man with sustained ventricular tachycardia. Echocardiography detected left ventricular (LV) contractile dysfunction with an ejection fraction of 49% and thinning of the LV wall (No.1, Online Resource 2). The maximum intensity projection image of positron emission tomography (PET) showed no abnormal ^18^F‐fluorodeoxyglucose (FDG) uptake other than the heart (A). The PET/CT fusion image revealed abnormal FDG uptake of focal-on-diffuse pattern in the left ventricular myocardium (B). Cardiovascular magnetic resonance showed late gadolinium enhancement, consistent with abnormal FDG uptake (C). There was no evidence of extracardiac sarcoidosis, and the patient was diagnosed with isolated cardiac sarcoidosis since he met the five major criteria

Regarding the myocardial FDG uptake pattern, 25 patients in the sCS group had focal uptake and 30 had focal-on-diffuse uptake. In contrast, in the iCS group, 8 patients had focal uptake and 19 had focal-on-diffuse uptake. There was no significant difference in the myocardial FDG uptake patterns between the two groups. However, the number of abnormal uptake segments was significantly higher in the iCS group than in the sCS group (10 [5–12] vs. 8 [4–10], *P* = 0.032). Two patients with iCS (7.4%) and 20 with sCS (36.4%) had RV uptake, and the frequency was significantly lower in the iCS group than in the sCS group (*P* = 0.0054). The myocardial SUVmax was significantly lower in the iCS group than in the sCS group (5.3 [4.6-9.4] vs. 9.8 [5.7-12.0], *P* = 0.0032). The TBR was also significantly lower in the iCS group than in the sCS group (3.6 [2.8-6.4] vs. 6.9 [3.8-8.8], *P* = 0.0010, Table 2). A representative case of iCS is shown in Figure 2.

**Table 2:** Positron emission tomography/computed tomography findings

	sCS (n = 55)	iCS (n = 27)	*P* value
Uptake pattern (focal/focal-on-diffuse)	25 (46.0%) / 30	8 (29.6%) / 19	0.17
Number of abnormal uptake segments	8 (4–10)	10 (5–12)	0.032
FDG uptake in the right ventricular myocardium	20 (36.4%)	2 (7.4%)	0.0054
SUVmax	9.8 (5.7–12.0)	5.3 (4.6–9.4)	0.0032
TBR	6.9 (3.8–8.8)	3.6 (2.8–6.4)	0.0010

Data are presented as median (interquartile range) or n (%)

*sCS*, systemic sarcoidosis with cardiac involvement; *iCS*, isolated cardiac sarcoidosis; *FDG*, ^18^F-fluorodeoxyglucose; *SUVmax*, maximum standardized uptake value; *TBR*, target-to-background ratio

### Prognosis

The median follow-up time was 39.5 (17.8–67.0) months (range, 1–135 months). In total, 24 patients (29.3%) experienced adverse events. Four patients died of cardiac causes, nine developed FVA, and 11 were hospitalized for heart failure. Seven patients (12.7%) with sCS experienced adverse events (two cardiac deaths, four FVA, and one hospitalization for heart failure), as did 17 (63.0%) with iCS (two cardiac deaths, five FVA, and ten hospitalizations for heart failure). In addition, 48 patients (87.3%) with sCS and 10 (37.0%) with iCS received corticosteroid therapy during the follow-up period, and the frequency significantly differed between the two groups (*P* < 0.0001). Furthermore, the number of patients who could start corticosteroid therapy with the LVEF maintained at ≥ 50% was significantly higher in 27 patients (49.1%) with sCS than in 3 (11.1%) with iCS (*P* = 0.0008).

**Figure 3:**
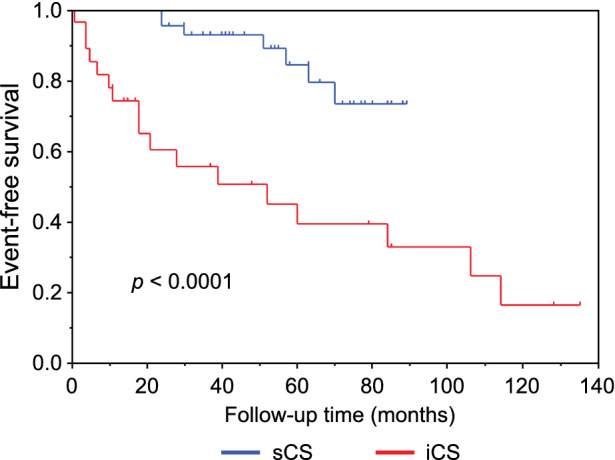
Kaplan–Meier curves for event-free survival in patients with isolated cardiac sarcoidosis or systemic sarcoidosis with cardiac involvement. The event-free survival rate was lower in the isolated cardiac sarcoidosis (iCS) group than in the systemic sarcoidosis with cardiac involvement (sCS) group (log-rank test, *P* < 0.0001)

Kaplan–Meier event-free survival curves for the two groups are presented in Figure 3. The event-free survival rate was lower in the iCS group than in the sCS group (log-rank test, *P* < 0.0001). Age, sex, CS type (iCS or sCS), the number of major criteria met (Online Resource 1), FVA as an initial symptom, LVEF < 50%, the presence of basal thinning of the interventricular septum, TBR, the number of abnormal uptake segments, the presence of FDG uptake in the RV myocardium, the rate of corticosteroid therapy during follow-up, and the presence of defibrillator implantation at baseline were analyzed using a univariate Cox proportional hazards model. CS type, LVEF < 50%, the rate of corticosteroid therapy during the follow-up period, and the number of abnormal FDG uptake segments were significantly associated with adverse events (Table 3). We selected CS type and the rate of corticosteroid therapy as variables for inclusion in the multivariate Cox proportional hazards model (Table 4), and iCS was found to be the only significant prognostic factor.

**Table 3:** Univariate cox proportional hazards model

Variable	Hazard ratio (95% CI)	*P* value
Age (years)	1.01 (0.97–1.05)	0.76
Male	1.43 (0.64–3.2)	0.39
iCS	5.27 (2.14–13.00)	0.0002
Number of major criteria met ^a^	1.40 (0.85–2.44)	0.20
FVA as an initial symptom	1.43 (0.62–3.30)	0.40
LVEF < 50%	4.16 (1.42–12.22)	0.003
Basal thinning of interventricular septum	0.96 (0.35–2.61)	0.94
TBR	0.94 (0.81–1.08)	0.39
Number of abnormal FDG uptake segments	1.14 (1.03–1.27)	0.010
FDG uptake in the right ventricular myocardium	0.55 (0.19–1.64)	0.26
Corticosteroid therapy during follow-up	0.27 (0.12–0.62)	0.0024
Defibrillator implantation at baseline	1.34 (0.60–3.00)	0.48

*iCS*, isolated cardiac sarcoidosis; *FVA*, fatal ventricular arrhythmia; *LVEF*, left ventricular ejection fraction; *TBR*, target-to-background ratio; *FDG*, ^18^F-fluorodeoxyglucose; *CI*, confidence interval

^a^Major criteria are in the “Diagnostic guidelines for cardiac sarcoidosis”^10^

**Table 4:** Multivariate cox proportional hazards model

Variable	Hazard ratio (95% CI)	*P* value
iCS	3.82 (1.42–10.30)	0.0059
Corticosteroid therapy during follow-up	0.47 (0.18–1.20)	0.11

*iCS*, isolated cardiac sarcoidosis; *CI*, confidence interval

## Discussion

The main finding of this study was that the event-free survival was lower in the iCS group than in the sCS group, and that iCS itself was an independent prognostic factor for adverse events in patients with CS. The JCS 2016 guidelines have established clinical diagnostic criteria for iCS in order to initiate prompt and appropriate treatment.^10^ We have suggested that iCS diagnosed using these criteria is an important prognostic predictor of cardiac events.

Our study included CS diagnosed by FDG PET/CT with a dedicated preparation protocol, i.e., a long fasting period along with a low-carbohydrate diet. In FDG PET/CT, physiological uptake in the myocardium has been a major concern in diagnosing CS; however, the combination of prolonged fasting and dietary restriction has made it possible to suppress physiological uptake and to correctly detect active inflammation.^13^ The first meta-analysis by Youssef et al.^20^ reported a diagnostic sensitivity of 89% and specificity of 78% for the diagnosis of CS by FDG PET. More recently, several studies have reported that the combination of an 18-hour fast and a low-carbohydrate diet protocol as a pretreatment increases specificity to ≥ 90%.^13,14^ Furthermore, FDG PET/CT is useful for the evaluation of systemic lesions, lesion activity, and the response to treatment. The establishment of this cardiac PET protocol was a major turning point in the clinical diagnosis of CS, ensuring the validity and relevance of the current guidelines for the diagnosis of iCS.

The frequency of iCS was 32.9% among patients included in this study. We carefully differentiated iCS by combining FDG PET/CT, CMR, clinical course, and other clinical findings. The 92.6% patients of iCS group underwent CMR and had LGE suggestive of CS.^18,19^ A previous review article reported that the prevalence of iCS among patients with CS varied widely due to the lack of diagnostic criteria for iCS, and the prevalence of iCS based on the available data in that review was approximately 25%.^21^ In recent reports, the prevalence of iCS diagnosed using the new JCS guidelines was 20.6–25.0%.^22,23^ One reason for the higher frequency of iCS in our study may be that patients receiving corticosteroid treatment for sCS before FDG PET/CT imaging were excluded. Furthermore, since cardiac symptoms suggestive of CS and positive findings on FDG PET examination are required for a diagnosis of iCS, it is possible that more iCS patients may not have been properly diagnosed than expected.

The number of male patients was significantly higher than the number of females in the iCS group than in the sCS. In two reports of iCS diagnosed by the same JCS 2016 guidelines, the proportion of male patients with iCS corresponded to 41%^22^ and 57%^23^ of the total study sample, indicating that there were no significant differences. Although the definition of iCS differs from that used in our study, the other two reports found that iCS was significantly more common in female patients than in males.^24,25^ Our findings that iCS was significantly more common in male patients than sCS, to the best of our knowledge, is the first of its kind, and further accumulation of cases is awaited.

ACE and sIL-2R levels were lower in the iCS group than in the sCS group. It has been reported that more patients with CS with extracardiac disease have elevated serum ACE, serum lysozyme, and urinary calcium excretion, suggesting a greater burden of granulomatous changes.^24^ It was similarly suspected that iCS had a lower burden of granulomatous changes than sCS in this study. The iCS group had a lower LVEF, higher number of abnormal uptake segments on PET, and lower TBR than did the sCS group. These results suggest that iCS is suspected to have more advanced scarring than sCS. iCS takes longer to diagnose because of the need for cardiac symptoms. In some patients, sCS was detected earlier by cardiac screening tests after the diagnosis of extracardiac lesions.

In 2011, Kandolin et al.^25^ reported that 81.8% of patients with iCS had LV systolic dysfunction and 51.5% developed adverse events. In 2015, the same group reported that patients with iCS had a higher rate of LV dysfunction and septal abnormalities on echocardiography and a higher prevalence of LGE on CMR, and iCS predicted a poorer event-free survival.^24^ These results suggest that iCS was detected at a more advanced stage. Similar to their results, iCS may have been diagnosed at an advanced stage in our patient population.

iCS was identified as an independent prognostic factor in the multivariate Cox proportional hazards model. The reason for the poor prognosis of iCS may be the lack of extracardiac sarcoid lesions, which makes early diagnosis difficult and delays the introduction of corticosteroid therapy. The iCS group was often not treated with corticosteroids, and only a few patients were started on treatment with preserved LVEF. The patients who did not receive corticosteroid therapy included those who could not be diagnosed with CS due to negative biopsies and those who had a low SUVmax and were diagnosed with insufficient active inflammation to warrant corticosteroid therapy.

To date, there is no consensus on the optimal treatment for iCS; therefore, treatment is the same as that for CS.^10,26^ In general, the prognosis of patients with CS is better when corticosteroid therapy is started before rather than after the occurrence of cardiac dysfunction.^8^ In the future, prospective studies on the effect of corticosteroid therapy for iCS are needed. We have suggested that iCS is an independent prognostic factor. It is necessary to further investigate the pathogenic differences between iCS and sCS.

Our iCS cohort had negative pathological results and was diagnosed according to clinical diagnostic criteria. Similarly, there was a previous study investigating the prognosis of presumed CS patients, who had unexplained high-grade AVB or ventricular arrhythmia, and suggestive CS findings for either CMR or FDG PET with negative pathology (including 61.4% iCS).^27^ In the study, they stated the presumed CS patients had a comparable high risk as those with histologic evidence and that the clinical categorization of presumed CS may be a useful tool to determine whether immunosuppression is effective. This study seems to support our results.

A limitation of this study includes the small number of patients. Although a multivariate Cox proportional hazards analysis was performed, the number of variables was limited due to the small number of adverse events. In addition, this was a retrospective study of consecutive patients diagnosed over 11 years, and the scanning equipment and conditions were not completely unified in all patients. Future prospective studies on the prognosis of iCS are warranted.

Exclusion of other diseases, such as dilated cardiomyopathy (DCM), arrhythmogenic cardiomyopathy, cardiac amyloidosis, tuberculous myocarditis, and hypertrophic cardiomyopathy, that may mimic iCS is an important and difficult problem.^28–32^ Parameters such as the coefficient of variation that are known to be useful for a distinction between iCS and DCM^28^ were not investigated in this study, which is one of the present limitations and an area for further investigation. In addition, the responsiveness of corticosteroid therapy to clinically diagnosed iCS may be important in diagnosis and treatment. Further prospective evaluation is needed.

## New knowledge gained

The number of abnormal uptake segments was significantly higher in the iCS group than in the sCS group, and the myocardial SUVmax and TBR were significantly lower in the iCS group than in the sCS group. iCS diagnosed using the new JCS guidelines was an independent prognostic factor for adverse cardiac events in patients with CS.

### Conclusion

Our study indicated that patients with iCS diagnosed using the JCS guidelines had a poorer prognosis than those with sCS, and iCS was an independent prognostic factor for adverse cardiac events. The reason for the poor prognosis of iCS may be the lack of extracardiac sarcoid lesions, which makes early diagnosis difficult and may delay the initiation of corticosteroid therapy. iCS needs to be recognized early, and a clinical diagnosis based on these new Japanese guidelines and FDG PET/CT with a dedicated preparation protocol may be helpful. Further investigation is warranted to characterize iCS and prospectively evaluate the outcomes and treatment effectiveness.

## Supplementary Information

Below is the link to the electronic supplementary material.

Supplementary file1 (PDF 190 kb)

Supplementary file2 (PDF 290 kb)

Supplementary file3 (PDF 253 kb)

Supplementary file4 (PPTX 600 kb)

Supplementary file5 (MP3 1861 kb)

## Abbreviations

CS: Cardiac sarcoidosis
iCS: Isolated cardiac sarcoidosis
sCS: Systemic sarcoidosis including cardiac involvement
JCS: Japanese Circulation Society
PET: Positron emission tomography
FDG: ^18^F-Fluorodeoxyglucose
SUV: Standardized uptake value
TBR: Target-to-background ratio
LV: Left ventricular
EF: Ejection fraction
