# Supplementary material for: Clinical features and prognosis of isolated cardiac sarcoidosis diagnosed using new guidelines with dedicated FDG PET/CT
Source: J Nucl Cardiol. 2022 Jul 8;30(1):280–9. doi: 10.1007/s12350-022-03034-0 (PMC9984349; doi:10.1007/s12350-022-03034-0)
Supplement: Supplementary file 1 — Supplementary file1 (PDF 190 kb) [file 12350_2022_3034_MOESM1_ESM.pdf]

Online Resource 1

Clinical features and prognosis of isolated cardiac sarcoidosis diagnosed using new guidelines with dedicated FDG PET/CT

Journal of Nuclear Cardiology

Tomohisa Okada [a], Naoto Kawaguchi [a], Masao Miyagawa [a]*, Marika Matsuoka [a], Rami Tashiro [a], Yuki Tanabe [a], Tomoyuki Kido [a], Toru Miyoshi [b], Haruhiko Higashi [b], Takeshi Inoue [c], Hideki Okayama [d], Osamu Yamaguchi [b], Teruhito Kido [a]

[a] Department of Radiology, Ehime University Graduate School of Medicine, Toon, Japan
[b] Department of Cardiology, Pulmonology, Hypertension and Nephrology, Ehime University Graduate School of Medicine, Toon, Japan
[c] Department of Radiology, Ehime Prefectural Central Hospital, Matsuyama, Japan
[d] Department of Cardiology, Ehime Prefectural Central Hospital, Matsuyama, Japan

*Corresponding author information: Masao Miyagawa, MD, PhD
E-mail: miyagawa@m.ehime-u.ac.jp

Online Resource 1 Diagnostic Guidelines for Cardiac Sarcoidosis and Isolated Cardiac Sarcoidosis

**Diagnostic Guidelines for Cardiac Sarcoidosis**
**Clinical findings defining cardiac involvement**
Cardiac findings should be assessed based on the major and the minor criteria. Clinical findings that satisfy the following 1) or 2) strongly suggest the presence of cardiac involvement.
1) Two or more of the five major criteria (a) to (e) are satisfied.
2) One in the five major criteria (a) to (e) and two or more of the three minor criteria (f) to (h) are satisfied.
**Criteria for cardiac involvement**
**1. Major criteria**
(a) High-grade atrioventricular block (including complete atrioventricular block) or fatal ventricular arrhythmia (e.g., sustained ventricular tachycardia and ventricular fibrillation)
(b) Basal thinning of the ventricular septum or abnormal ventricular wall anatomy (ventricular aneurysm, thinning of the middle or upper ventricular septum, regional ventricular wall thickening)
(c) Left ventricular contractile dysfunction (left ventricular ejection fraction less than 50%) or focal ventricular wall asynergy
(d) ^{67}Ga citrate scintigraphy or ^{18}F-FDG PET reveals abnormally high tracer accumulation in the heart
(e) Gadolinium-enhanced MRI reveals delayed contrast enhancement of the myocardium
**2. Minor criteria**
(f) Abnormal ECG findings: Ventricular arrhythmias (nonsustained ventricular tachycardia, multifocal or frequent premature ventricular contractions), bundle branch block, axis deviation, or abnormal Q waves
(g) Perfusion defects on myocardial perfusion scintigraphy (SPECT)
(h) Endomyocardial biopsy: Monocyte infiltration and moderate or severe myocardial interstitial fibrosis
**Diagnostic guidelines for cardiac sarcoidosis**
1) Histological diagnosis group (those with positive myocardial biopsy findings)
Cardiac sarcoidosis is diagnosed histologically when endomyocardial biopsy or surgical specimens demonstrate non-caseating epithelioid granulomas.
2) Clinical diagnosis group (those with negative myocardial biopsy findings or those not undergoing myocardial biopsy)
The patient is clinically diagnosed as cardiac sarcoidosis (1) when epithelioid granulomas are found in organs other than the heart, and clinical findings strongly suggestive of the above-mentioned cardiac involvement are present; or (2) when the patient shows clinical findings strongly suggestive of pulmonary or ophthalmic sarcoidosis; at least 2 of the five following characteristic laboratory findings of sarcoidosis (bilateral hilar lymphadenopathy: high serum ACE activity or elevated serum lysozyme levels: high serum sIL-2R levels: significant tracer accumulation in ^{67}Ga citrate scintigraphy or ^{18}F-FDG PET: a high percentage of lymphocytes with a CD4/CD8 ratio of >3.5 in BAL fluid); and clinical findings strongly suggest the above-mentioned cardiac involvement.
**Diagnostic Guidelines for Isolated Cardiac Sarcoidosis**
**Prerequisite**

1. No clinical findings characteristics of sarcoidosis are observed in any organs other than the heart (The patient should be examined in detail for respiratory, ophthalmic, and skin involvements of sarcoidosis. When the patient is symptomatic, other etiologies that can affect the corresponding organs must be ruled out.).
2. ^{67}Ga scintigraphy or ^{18}F-FDG PET reveals no abnormal tracer accumulation in any organs other than the heart.
3. A chest CT scan reveals no shadow along the lymphatic tracts in the lungs or no hilar and mediastinal lymphadenopathy (minor axis >10 mm).
**1) Histological diagnosis group**
Isolated cardiac sarcoidosis is diagnosed histologically when endomyocardial biopsy or surgical specimens demonstrate non-caseating epithelioid granulomas.
**2) Clinical diagnosis group**
Isolated cardiac sarcoidosis is diagnosed clinically when the criterion (d) and at least three other criteria of the major criteria (a) to (e) are satisfied.

This table was reprinted with permission from Circulation Journal.[1]

^{18}F-FDG PET, ^{18}F-fluorodeoxyglucose positron emission tomography; MRI, magnetic resonance imaging; ECG, electrocardiography; SPECT, single photon emission computed tomography; ACE, angiotensin-converting enzyme; sIL-2R, soluble interleukin-2 receptor; CD, cluster of differentiation; BAL, bronchoalveolar lavage; CT, computed tomography

Reference of the Online Resource 1

1. Terasaki F, Azuma A, Anzai T, Ishizaka N, Ishida Y, Isobe M, et al. JCS 2016 guideline on diagnosis and treatment of cardiac sarcoidosis — Digest version — Circ J 2019;83:2329–88.
