# Supplementary material for: Clinical features and prognosis of isolated cardiac sarcoidosis diagnosed using new guidelines with dedicated FDG PET/CT
Source: J Nucl Cardiol. 2022 Jul 8;30(1):280–9. doi: 10.1007/s12350-022-03034-0 (PMC9984349; doi:10.1007/s12350-022-03034-0)
Supplement: Supplementary file 2 — Supplementary file2 (PDF 290 kb) [file 12350_2022_3034_MOESM2_ESM.pdf]

Online Resource 2

Clinical features and prognosis of isolated cardiac sarcoidosis diagnosed using new guidelines with dedicated FDG PET/CT

Journal of Nuclear Cardiology

Tomohisa Okada [a], Naoto Kawaguchi [a], Masao Miyagawa [a*], Marika Matsuoka [a], Rami Tashiro [a], Yuki Tanabe [a], Tomoyuki Kido [a], Toru Miyoshi [b], Haruhiko Higashi [b], Takeshi Inoue [c], Hideki Okayama [d], Osamu Yamaguchi [b], Teruhito Kido [a]

[a] Department of Radiology, Ehime University Graduate School of Medicine, Toon, Japan
[b] Department of Cardiology, Pulmonology, Hypertension and Nephrology, Ehime University Graduate School of Medicine, Toon, Japan
[c] Department of Radiology, Ehime Prefectural Central Hospital, Matsuyama, Japan
[d] Department of Cardiology, Ehime Prefectural Central Hospital, Matsuyama, Japan

*Corresponding author information: Masao Miyagawa, MD, PhD
E-mail: miyagawa@m.ehime-u.ac.jp

Online Resource 2 Characteristics of Patients with Isolated Cardiac Sarcoidosis

No	Age	Sex	SUVmax	TBR	Cardiac uptake pattern	LVEF (%)	Adverse events	Event-free time (month)	Symptoms	Major criteria [a]	Endomyocardial biopsy	Corticosteroid therapy during follow-up	Cardiac uptake on follow-up FDG PET/CT
1	53	M	4.6	3.3	Focal-on-diffuse	49	FVA	18	VT, thinning of LV wall, and LV contractile dysfunction	a, b, c, d, e	Fibrosis	-	Decrease, then increase
2	61	M	6.2	4.8	Focal-on-diffuse	48	FVA	84	VT and LV contractile dysfunction	a, c, d, e	Fibrosis	-	Decrease
3	61	F	4.9	3.3	Focal-on-diffuse	36	Heart failure	10	Heart failure, thinning of LV wall, and AF	b, c, d, e	Cardiomyocyte degeneration	+	-
4	68	M	5.2	3.7	Focal-on-diffuse	47	Heart failure	60	VT, thickening of LV wall, and LV contractile dysfunction	a, b, c, d, e	Monocyte infiltration	-	-
5	76	M	3.5	2.7	Focal-on-diffuse	45	FVA	4	VT, thinning of LV wall, and LV contractile dysfunction	a, b, c, d, e	Cardiomyocyte degeneration	-	-
6	75	M	4.4	2.9	Focal	29	Heart failure	18	Heart failure, AFL, and thinning of LV wall	b, c, d, e	Monocyte infiltration	-	Decrease
7	63	M	4.7	2.5	Focal-on-diffuse	38	-	135	AVB, thinning of LV wall, and LV contractile dysfunction	a, b, c, d	Fibrosis	+	Decrease, then increase
8	64	M	4.5	2.4	Focal-on-diffuse	54	Cardiac death	21	AVB, thinning of LV wall, LV contractile dysfunction, and CRBBB	a, b, c, d, e	Monocyte infiltration	+	Decrease
9	55	F	9.6	6.4	Focal	58	-	128	AVB, thinning of LV wall, LV contractile dysfunction, CRBBB, and NSVT	a, b, c, d, e	No special findings	+	-
10	76	F	4.8	2.8	Focal-on-diffuse	30	Heart failure	5	Heart failure and thinning of LV wall	b, c, d, e	-	-	-
11	62	F	5.3	3.1	Focal-on-diffuse	60	FVA	7	VT, AVB, ventricular aneurysm, and abnormal LV motion	a, b, c, d	Monocyte infiltration	-	Mixture of increase and decrease
12	40	M	5.5	3.7	Focal-on-diffuse	28	Cardiac death	114	Heart failure, thinning of LV wall, and LV contractile dysfunction	b, c, d, e	Fibrosis	+	Increase
13	68	M	6.6	4.4	Focal-on-diffuse	37	Heart failure	28	Heart failure, thinning of LV wall, and AF	b, c, d, e	-	-	-
14	65	M	6.0	4.0	Focal-on-diffuse	54	-	48	AVB and LV contractile dysfunction	a, c, d, e	-	-	Decrease
15	69	F	7.5	6.8	Focal-on-diffuse	35	-	37	Heart failure, thinning of LV wall, and NSVT	b, c, d, e	No special findings	-	Decrease
16	62	M	2.4	1.8	Focal	53	-	17	VF, thin LV wall, and LV contractile dysfunction	a, b, c, d, e	Fibrosis	-	-
17	75	F	11.8	6.9	Focal-on-diffuse	34	Heart failure	1	Heart failure, thinning of LV wall, CLBBB, and AF	b, c, d, e	-	-	-
18	71	M	3.3	2.2	Focal	41	-	14	Heart failure and thinning of LV wall	b, c, d, e	Fibrosis	-	-
19	71	M	5.2	2.9	Focal	62	-	15	AVB, heart failure, thinning of LV wall, and CRBBB	a, b, c, d, e	No special findings	+	Decrease
20	42	F	10.1	6.7	Focal-on-diffuse	45	-	11	Thinning of LV wall and LV contractile dysfunction	b, c, d, e	-	-	-
21	59	F	15.0	10.0	Focal-on-diffuse	42	Heart failure	106	VT, AVB, thinning of LV wall, and LV contractile dysfunction	a, b, c, d, e	No special findings	+	No change
22	64	M	12.1	9.3	Focal	24	-	85	VT, AVB, heart failure, thin LV wall, and CLBBB	a, c, d, e	Cardiomyocyte degeneration	+	Mixture of increase and decrease
23	66	M	9.4	5.2	Focal-on-diffuse	34	Heart failure	11	Heart failure, thickening of LV wall, and CLBBB	b, c, d, e	Fibrosis	+	Decrease, then increase
24	62	M	5.0	2.9	Focal	35	Heart failure	39	Heart failure and thinning of LV wall	b, c, d, e	Monocyte infiltration	-	-
25	42	M	5.8	3.6	Focal-on-diffuse	50	-	79	Heart failure, thinning of LV wall, and LV contractile dysfunction	b, c, d, e	Details unknown	-	-
26	53	M	3.0	2.3	Focal	40	FVA	52	VT and LV contractile dysfunction	a, c, d, e	Fibrosis	-	No change
27	66	M	11.3	8.7	Focal-on-diffuse	34	Heart failure	4	Heart failure and thinning of LV wall	b, c, d, e	-	+	Decrease

[a] Major criteria are in the “Diagnostic guidelines for cardiac sarcoidosis” (a: high-grade atrioventricular block or fatal ventricular arrhythmia; b: basal thinning of the ventricular septum or abnormal

ventricular wall anatomy; c: left ventricular contractile dysfunction; d: ^{67}Ga-citrate scintigraphy or ^{18}F-fluorodeoxyglucose positron emission tomography reveals abnormally high tracer accumulation in the heart; e: Gadolinium-enhanced magnetic resonance imaging reveals delayed contrast enhancement of the myocardium).[1]

iCS, isolated cardiac sarcoidosis; M, male; F, female; SUVmax, maximum standardized uptake value; TBR, target-to-background ratio; FVA, fatal ventricular arrhythmia; LVEF, left ventricular ejection fraction; VT, ventricular tachycardia; LV, left ventricular; AF, atrial fibrillation; AFL, atrial flutter; AVB, atrioventricular block; NSVT, non-sustained ventricular tachycardia; CRBBB, complete right bundle branch block; CLBBB, complete left bundle branch block; FDG PET/CT, ^{18}F-fluorodeoxyglucose positron emission tomography/computed tomography

Reference of the Online Resource 2

1. Terasaki F, Azuma A, Anzai T, Ishizaka N, Ishida Y, Isobe M, et al. JCS 2016 guideline on diagnosis and treatment of cardiac sarcoidosis — Digest version — Circ J 2019;83:2329–88.
