# Supplementary material for: Clinical features and prognosis of isolated cardiac sarcoidosis diagnosed using new guidelines with dedicated FDG PET/CT
Source: J Nucl Cardiol. 2022 Jul 8;30(1):280–9. doi: 10.1007/s12350-022-03034-0 (PMC9984349; doi:10.1007/s12350-022-03034-0)

Clinical features and prognosis of isolated cardiac sarcoidosis diagnosed using new guidelines with dedicated FDG PET/CT

Diagnostic guidelines for isolated cardiac sarcoidosis (iCS) were first proposed in 2016. This study aimed to evaluate the use of ^{18}F-fluorodeoxyglucose positron emission tomography/computed tomography (FDG PET/CT) imaging in predicting iCS prognosis.

We included 82 patients (55 with systemic sarcoidosis including cardiac involvement [sCS], 27 with iCS) in the study. We compared the FDG PET/CT findings between the two groups. We examined the relationship between the CS type and the rate of adverse cardiac events.

iCS was an independent prognostic factor for adverse cardiac events in patients with CS. The clinical diagnosis of iCS based on FDG PET/CT and new guidelines may be important.

Table: PET/CT finding

	sCS (n = 55)	iCS (n = 27)	p value
Uptake pattern (focal/focal-on-diffuse)	25 / 30	8 / 19	0.17
Number of abnormal uptake segments	8 (4–10)	10 (5–12)	0.032
FDG uptake in the right ventricular myocardium	20 (36.4%)	2 (7.4%)	0.0054
SUVmax	9.8 (5.7–12.0)	5.3 (4.6–9.4)	0.0032
Target to background ratio	6.9 (3.8–8.8)	3.6 (2.8–6.4)	0.0010

Figure: Kaplan–Meier curves for event-free survival

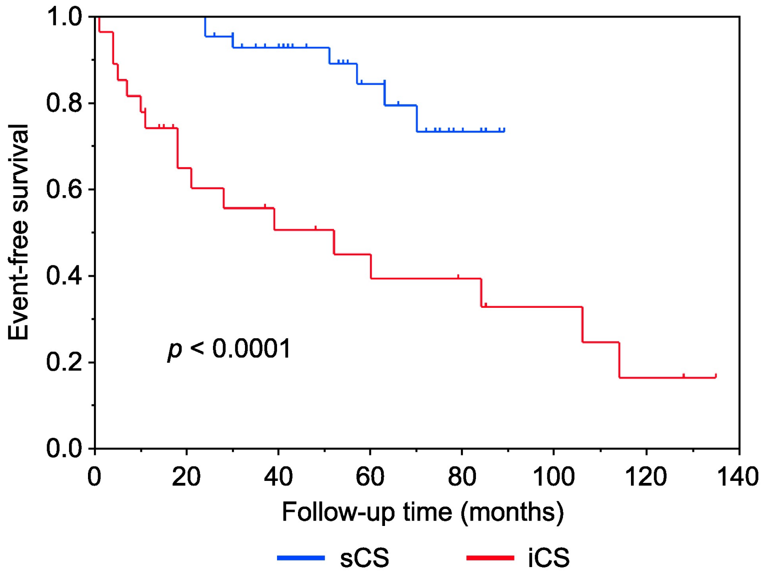

Supplement: Supplementary file 3 — Supplementary file3 (PDF 253 kb) [file 12350_2022_3034_MOESM3_ESM.pdf]
