# Supplementary material for: Clinical features and prognosis of isolated cardiac sarcoidosis diagnosed using new guidelines with dedicated FDG PET/CT
Source: J Nucl Cardiol. 2022 Jul 8;30(1):280–9. doi: 10.1007/s12350-022-03034-0 (PMC9984349; doi:10.1007/s12350-022-03034-0)
Supplement: Supplementary file 4 — Supplementary file4 (PPTX 600 kb) [file 12350_2022_3034_MOESM4_ESM.pptx]

## Slide 1
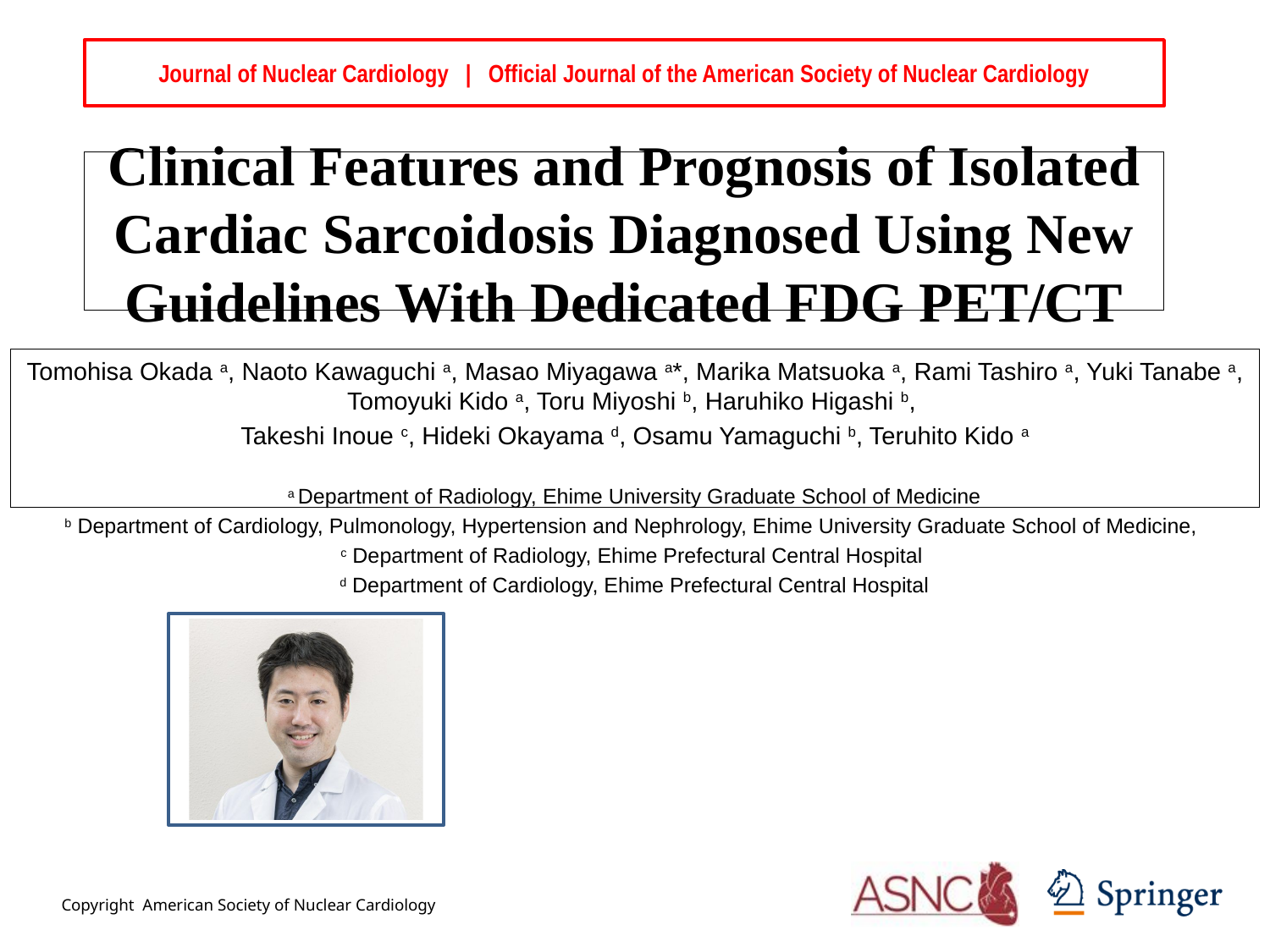

Journal of Nuclear Cardiology | Official Journal of the American Society of Nuclear Cardiology
# Clinical Features and Prognosis of Isolated Cardiac Sarcoidosis Diagnosed Using New Guidelines With Dedicated FDG PET/CT
Tomohisa Okada a, Naoto Kawaguchi a, Masao Miyagawa a*, Marika Matsuoka a, Rami Tashiro a, Yuki Tanabe a, Tomoyuki Kido a, Toru Miyoshi b, Haruhiko Higashi b,
Takeshi Inoue c, Hideki Okayama d, Osamu Yamaguchi b, Teruhito Kido a
a Department of Radiology, Ehime University Graduate School of Medicine
b Department of Cardiology, Pulmonology, Hypertension and Nephrology, Ehime University Graduate School of Medicine,
c Department of Radiology, Ehime Prefectural Central Hospital
d Department of Cardiology, Ehime Prefectural Central Hospital
Head shot of author
required
Copyright American Society of Nuclear Cardiology

## Slide 2
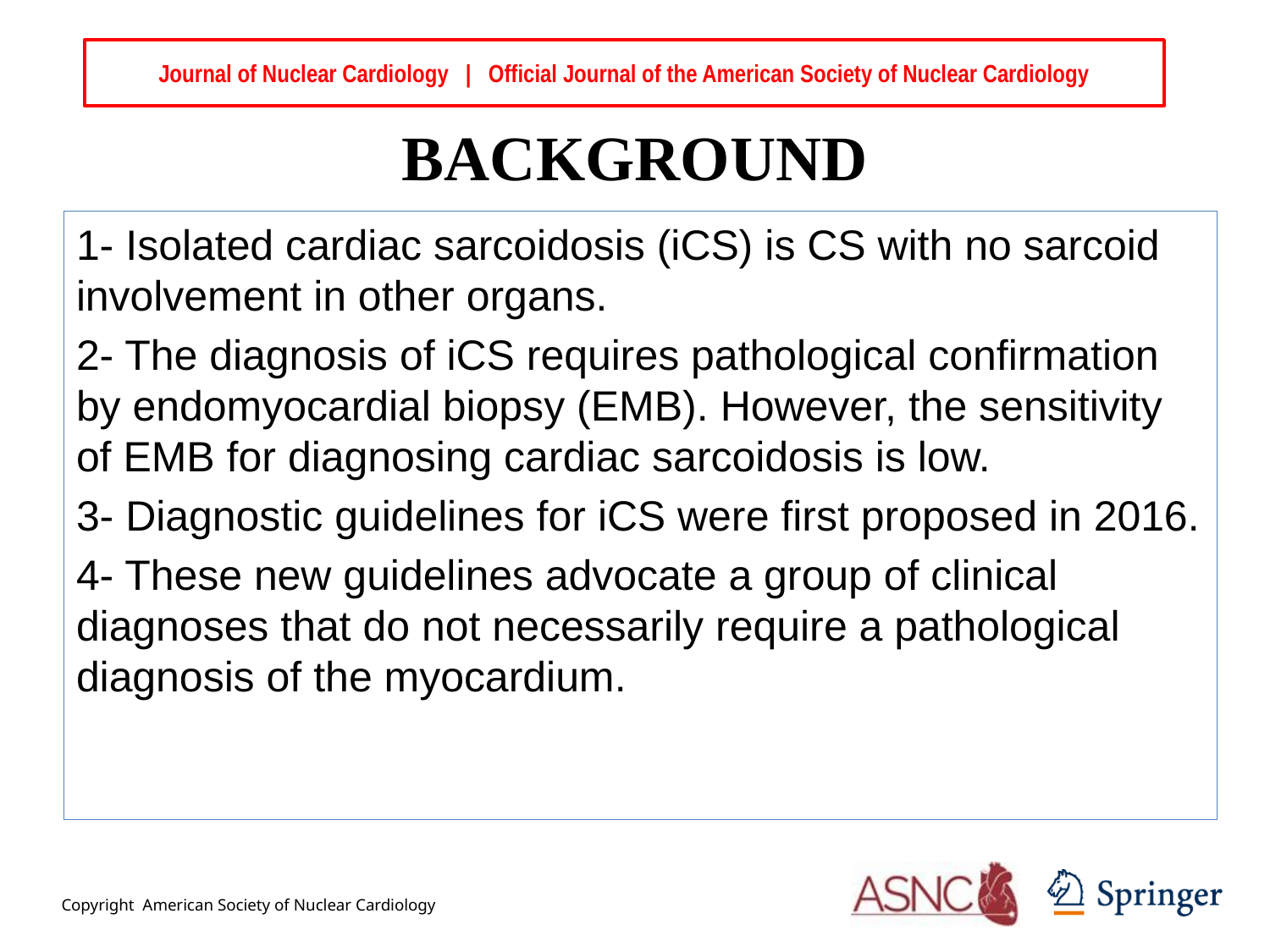

Journal of Nuclear Cardiology | Official Journal of the American Society of Nuclear Cardiology
# BACKGROUND
1- Isolated cardiac sarcoidosis (iCS) is CS with no sarcoid involvement in other organs.
2- The diagnosis of iCS requires pathological confirmation by endomyocardial biopsy (EMB). However, the sensitivity of EMB for diagnosing cardiac sarcoidosis is low.
3- Diagnostic guidelines for iCS were first proposed in 2016.
4- These new guidelines advocate a group of clinical diagnoses that do not necessarily require a pathological diagnosis of the myocardium.
Copyright American Society of Nuclear Cardiology

## Slide 3
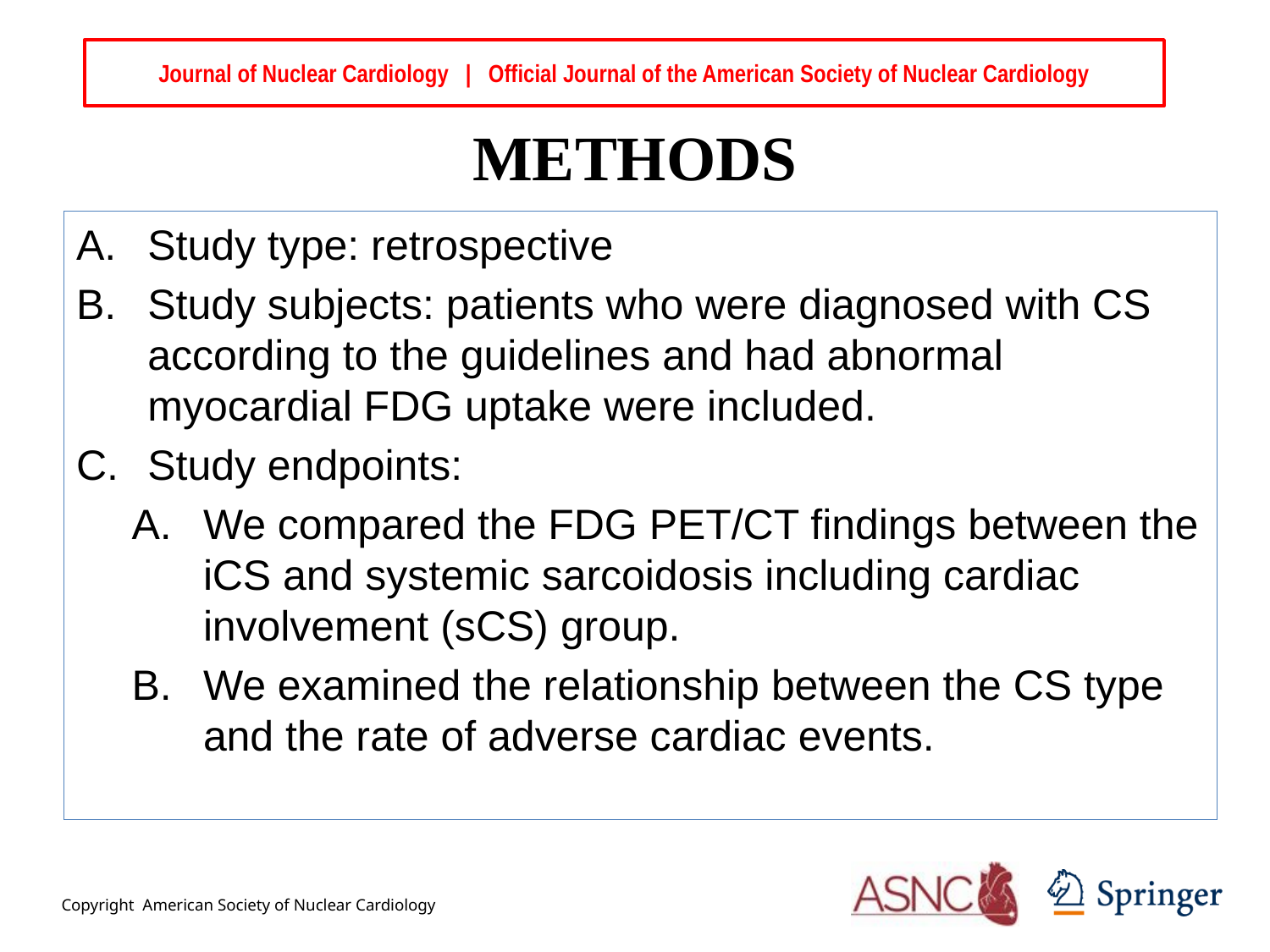

Journal of Nuclear Cardiology | Official Journal of the American Society of Nuclear Cardiology
# METHODS
Study type: retrospective
Study subjects: patients who were diagnosed with CS according to the guidelines and had abnormal myocardial FDG uptake were included.
Study endpoints:
We compared the FDG PET/CT findings between the iCS and systemic sarcoidosis including cardiac involvement (sCS) group.
We examined the relationship between the CS type and the rate of adverse cardiac events.
Copyright American Society of Nuclear Cardiology

## Slide 4
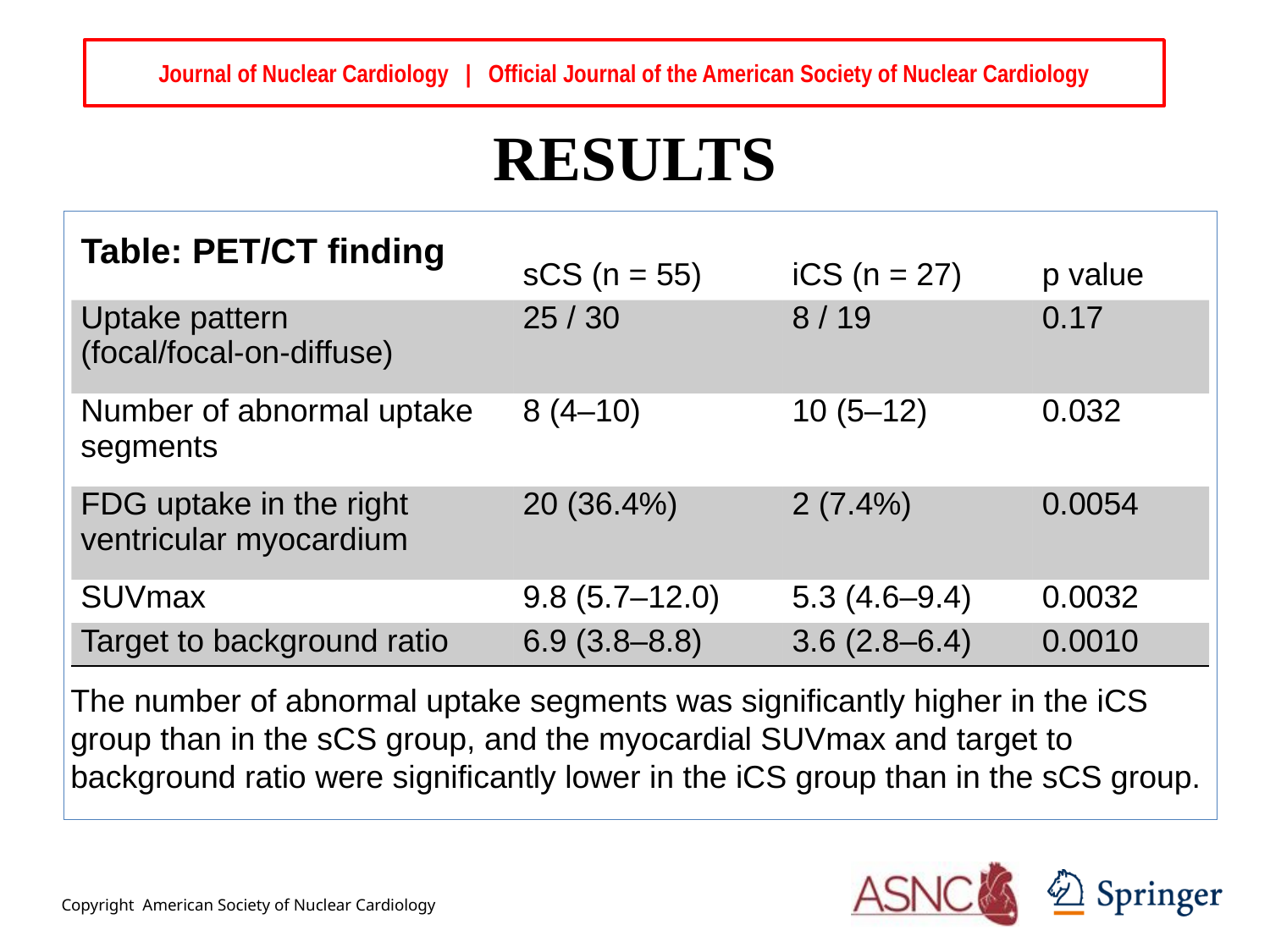

Journal of Nuclear Cardiology | Official Journal of the American Society of Nuclear Cardiology
# RESULTS
Table: PET/CT finding
| | sCS (n = 55) | iCS (n = 27) | p value |
| --- | --- | --- | --- |
| Uptake pattern (focal/focal-on-diffuse) | 25 / 30 | 8 / 19 | 0.17 |
| Number of abnormal uptake segments | 8 (4–10) | 10 (5–12) | 0.032 |
| FDG uptake in the right ventricular myocardium | 20 (36.4%) | 2 (7.4%) | 0.0054 |
| SUVmax | 9.8 (5.7–12.0) | 5.3 (4.6–9.4) | 0.0032 |
| Target to background ratio | 6.9 (3.8–8.8) | 3.6 (2.8–6.4) | 0.0010 |
The number of abnormal uptake segments was significantly higher in the iCS group than in the sCS group, and the myocardial SUVmax and target to background ratio were significantly lower in the iCS group than in the sCS group.
Copyright American Society of Nuclear Cardiology

## Slide 5
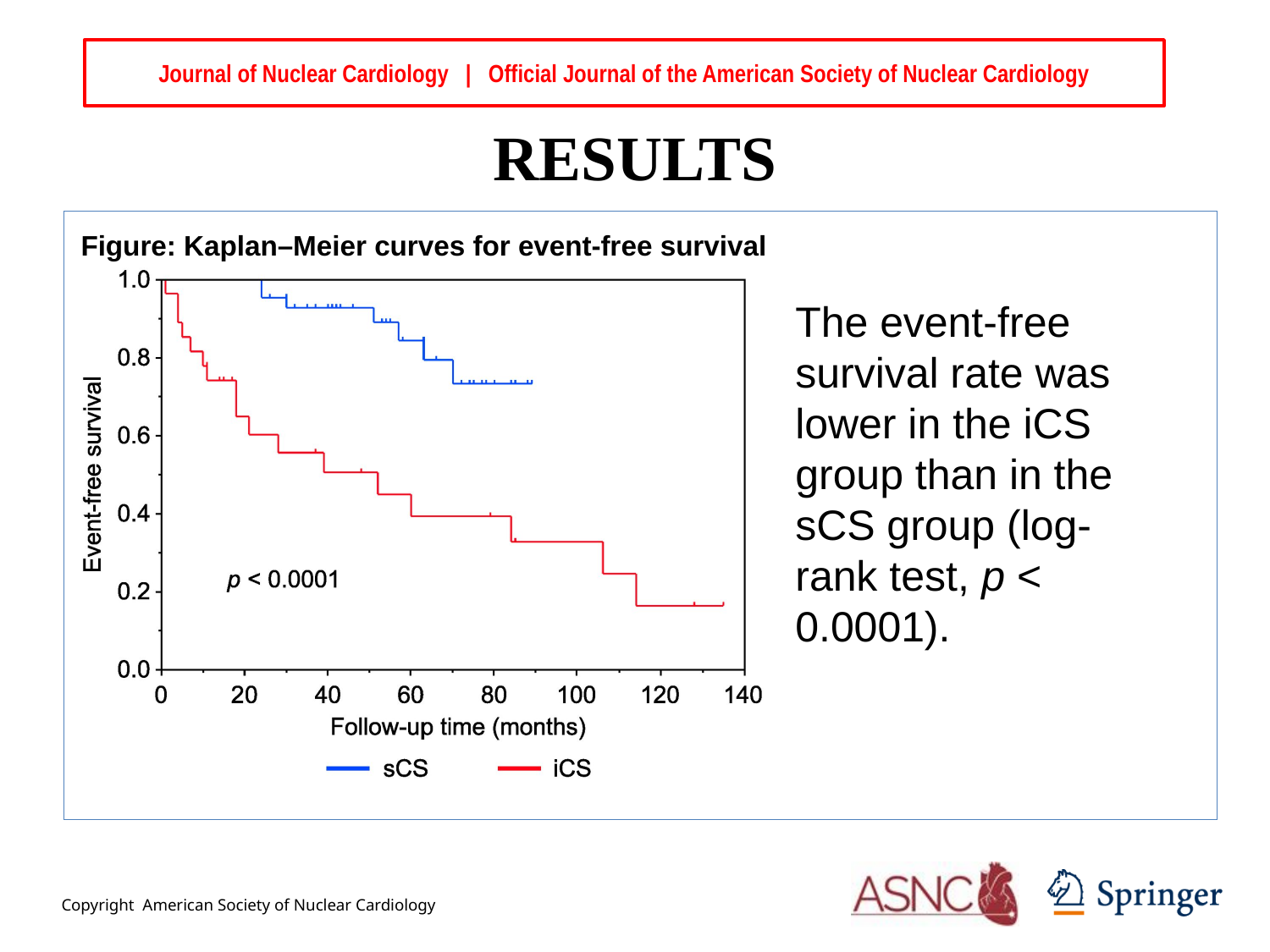

Journal of Nuclear Cardiology | Official Journal of the American Society of Nuclear Cardiology
# RESULTS
Figure: Kaplan–Meier curves for event-free survival
The event-free survival rate was lower in the iCS group than in the sCS group (log-rank test, p < 0.0001).
Copyright American Society of Nuclear Cardiology

## Slide 6
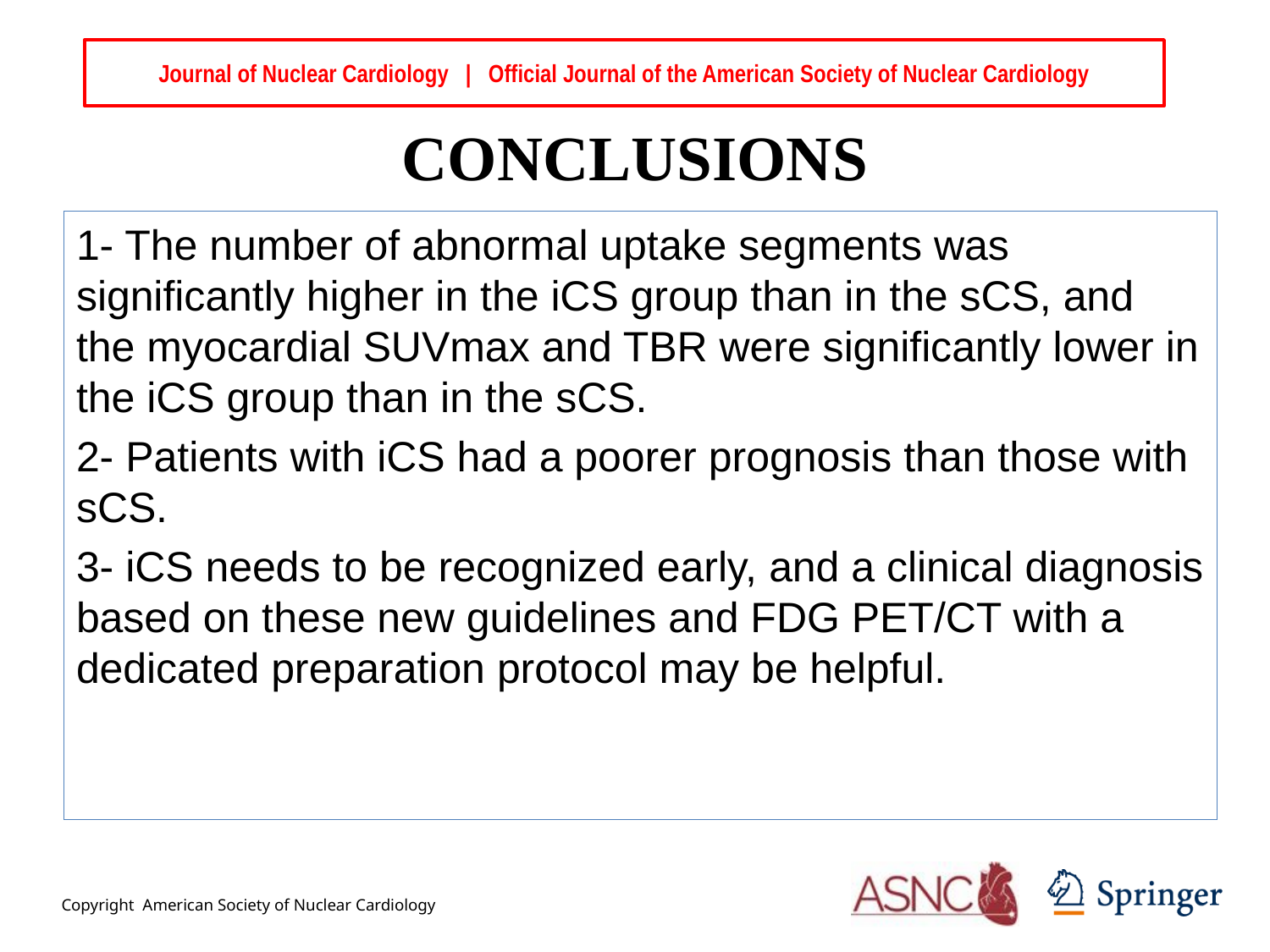

Journal of Nuclear Cardiology | Official Journal of the American Society of Nuclear Cardiology
# CONCLUSIONS
1- The number of abnormal uptake segments was significantly higher in the iCS group than in the sCS, and the myocardial SUVmax and TBR were significantly lower in the iCS group than in the sCS.
2- Patients with iCS had a poorer prognosis than those with sCS.
3- iCS needs to be recognized early, and a clinical diagnosis based on these new guidelines and FDG PET/CT with a dedicated preparation protocol may be helpful.
Copyright American Society of Nuclear Cardiology
